# Leukotriene signaling as molecular correlate for cognitive heterogeneity in aging: an exploratory study

**DOI:** 10.3389/fnagi.2023.1140708

**Published:** 2023-08-02

**Authors:** Heike Mrowetz, Mohamed H. Kotob, Jennifer Forster, Iren Aydin, Michael Stefan Unger, Jana Lubec, Ahmed M. Hussein, Jovana Malikovic, Daniel Daba Feyissa, Volker Korz, Harald Höger, Gert Lubec, Ludwig Aigner

**Affiliations:** ^1^Institute of Molecular Regenerative Medicine, Paracelsus Medical University, Salzburg, Austria; ^2^Spinal Cord Injury and Tissue Regeneration Center Salzburg (SCI-TReCS), Paracelsus Medical University, Salzburg, Austria; ^3^Programme for Proteomics, Paracelsus Medical University, Salzburg, Austria; ^4^Department of Pathology, Faculty of Veterinary Medicine, Assiut University, Assiut, Egypt; ^5^Department of Pharmaceutical Sciences, Division of Pharmaceutical Chemistry, Faculty of Life Sciences, University of Vienna, Vienna, Austria; ^6^Department of Zoology, Faculty of Science, Al-Azhar University, Asyut, Egypt; ^7^Core Unit of Biomedical Research, Division of Laboratory Animal Science and Genetics, Medical University of Vienna, Himberg, Austria; ^8^Austrian Cluster for Tissue Regeneration, Vienna, Austria

**Keywords:** cognition, aging, microglia, 5-LOX, FLAP

## Abstract

**Introduction:**

Aging is in general associated with a decline in cognitive functions. Looking more closely, there is a huge heterogeneity in the extent of cognitive (dys-)abilities in the aged population. It ranges from the population of resistant, resilient, cognitively unimpaired individuals to patients with severe forms of dementias. Besides the known genetic, environmental and life style factors that shape the cognitive (dys-)abilities in aging, the underlying molecular mechanisms and signals related to cognitive heterogeneity are completely unknown. One putative mechanism underlying cognitive heterogeneity might be neuroinflammation, exerted through microglia, the brain’s innate immune cells, as neuroinflammation is central to brain aging and neurodegenerative diseases. Recently, leukotrienes (LTs), i.e., small lipid mediators of inflammation produced by microglia along aging and neurodegeneration, got in the focus of geroscience as they might determine cognitive dysfunctions in aging.

**Methods:**

Here, we analyzed the brain’s expression of key components of the LT synthesis pathway, i.e., the expression of 5-lipoxygenase (5-Lox), the key enzyme in LT production, and 5-lipoxygenase-activating protein (FLAP) in young and aged rats. More specifically, we used a cohort of rats, which, although grown up and housed under identical conditions, developed into aged cognitively unimpaired and aged cognitively impaired traits.

**Results:**

Expression of 5-Lox was increased within the brain of aged rats with the highest levels detected in cognitively impaired animals. The number of microglia cells was higher in the aged compared to the young brains with, again, the highest numbers of 5-Lox expressing microglia in the aged cognitively impaired rats. Remarkably, lower cognitive scores in the aged rats associated with higher numbers of 5-Lox positive microglia in the animals. Similar data were obtained for FLAP, at least in the cortex. Our data indicate elevated levels of the LT system in the brain of cognitively impaired animals.

**Discussion:**

We conclude that 5-Lox expressing microglia potentially contribute to the age-related cognitive decline in the brain, while low levels of the LT system might indicate and foster higher cognitive functions and eventually cognitive reserve and resilience in aging.

## 1. Introduction

Physiological aging is associated with a decline in cognitive functions such as memory, executive function and processing information (reviewed in Zec, 1995; Harada et al., 2013). However, in a more detailed view, there is a huge heterogeneity in terms of cognitive abilities among the elderly population, which ranges from cognitively unimpaired, resistant and/or resilient individuals to patients with severe forms of dementias. This is not limited to humans, as a broad spectrum of cognitive heterogeneity develops for example also in aging rodents. While growing up in identical and controlled environmental conditions, i.e., standard animal facility conditions, Sprague-Dawley rats develop traits of aged cognitively unimpaired (AU) and aged cognitively impaired (AI) animals, which can be discriminated according their performance in cognitive behavior tests (Brouillette and Quirion, 2008; Farso et al., 2013; Lubec et al., 2019). Multiple changes in brain homeostasis can contribute to this age-related differences of cognitive abilities and are summarized as hallmarks of the aging brain including mitochondrial dysfunction, impaired molecular waste disposal, impaired DNA repair mechanisms, aberrant network activity, stem cell exhaustion, oxidative damage, dysregulated neuronal calcium homeostasis, impaired adaptive stress responses and glia cell activation and neuroinflammation (reviewed in Mattson and Arumugam, 2018).

Neuroinflammation is a complex process and a major contributor to brain aging as well as to various age-related neurodegenerative central nervous system (CNS) diseases, such as dementias including Alzheimer’s disease (AD), and movement disorders such as Parkinson’s disease (PD) (reviewed in Wyss-Coray and Mucke, 2002; Wyss-Coray, 2006; Heneka et al., 2015). Neuroinflammation is mediated by innate immune cells of the brain, i.e., the microglia cells (reviewed in Kettenmann et al., 2011; Kofler and Wiley, 2011). The main function of microglia is maintaining brain homeostasis and immune surveillance (reviewed in Aguzzi et al., 2013). In the elderly brain, microglia cells become pro-inflammatory and dysfunctional in their homeostatic properties (Delage et al., 2021), proteostasis and phagocytosis (Sierra et al., 2007; Marschallinger et al., 2020b, reviewed in Mosher and Wyss-Coray, 2014). Whether cognitive heterogeneity in aging is related to neuroinflammation, more specifically to microglia (dys-)functions is largely unexplored.

The LT signaling pathway is a prominent inflammatory pathway that is mainly known from its contribution to asthma in the lung (reviewed in Samuelsson, 1983; Okunishi and Peters-Golden, 2011). It has moved more recently in the focus of aging research, in particular in brain aging, since it was shown that leukotrienes are increased in the aging brain (Uz et al., 1998; Qu et al., 2000; Chinnici et al., 2007), and the leukotriene pathway has been suggested as a therapeutic target to rejuvenate the aged brain (McGilvray, 2016). Leukotrienes for example contribute to brain vessel damage (Peters-Golden, 2008; Marschallinger et al., 2015) and to microglia activation (Yu et al., 2014). Moreover, we demonstrated that in the brain, microglia are the main production site of leukotrienes, they harbor key components of the LT production pathway, i.e., 5-Lox and FLAP (Michael et al., 2020). Most importantly, we had shown that Montelukast (MTK), a leukotriene receptor antagonist and approved anti-asthma medication, reduces neuroinflammation, promotes neurogenesis and blood-brain-barrier integrity, and restores cognition in aged rats (Marschallinger et al., 2015).

Here, we extend our previous studies and used an aged cohort of rats modeling cognitive heterogeneity in aging. Animals were grouped in “good” old (Aged cognitively Unimpaired, AU) and “bad” old (Aged cognitively Impaired, AI) learners depending on their performance in the hole-board behavior test, a test for spatial learning and memory (Brouillette and Quirion, 2008; Farso et al., 2013; Lubec et al., 2019). We used brains from this previously described cohort to study molecular alterations in neuroinflammation related to the LT signaling pathway. We performed detailed immunohistochemical analysis of microglia (Iba1 positive cells) and LT signaling related proteins (5-Lox, FLAP) in the brains of young (Y), AU and AI rats.

## 2. Materials and methods

### 2.1. Animals

All animals used in this study were bred and maintained in the Core Unit of Biomedical Research, Division of Laboratory Animal Science and Genetics, Medical University of Vienna. Rats were housed in groups of two in standard Makrolon cages filled with autoclaved woodchips (temperature: 22 ± 2°C; humidity: 55 ± 5%; 12 h artificial light/12 h dark cycle: light on at 7:00 a.m.). Aged rats were on a low-calory diet (ssniff, R/M-H Ered II, Soest, Germany) from the fourth month of age. Tap water and food was provided *ad libitum*. All behavioral experiments were performed during the light phase of the light–dark cycle. One week prior and throughout the experiment, the animals lived individually in standard cages in a separate experimental room.

### 2.2. Hole-board spatial learning and memory test

**FIGURE 1:**
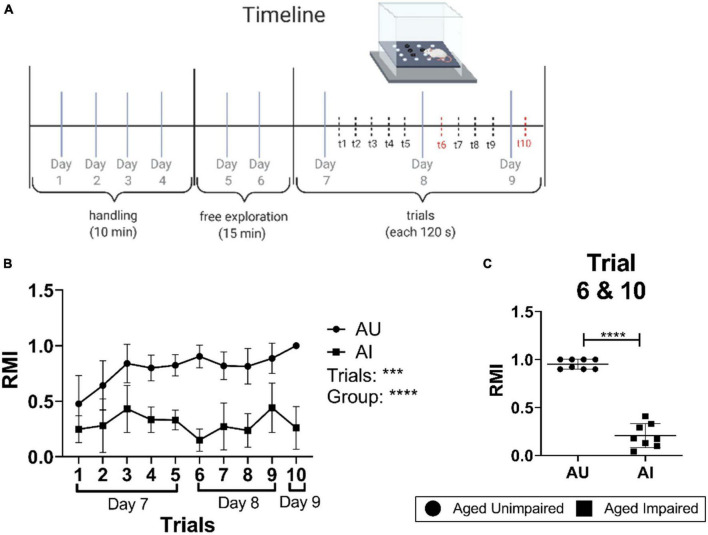
Experimental setting and performance in the hole-board spatial memory behavior test. **(A)** Scheme and time line of the hole-board spatial memory test. **(B)** Learning curves of AU and AI rats in the hole-board test showing mean RMI values of rats for each trial (*n* = 8–10/group). A Two-way RM-ANOVA with Sidak’s multiple comparison test was used to determine significant differences in RMI values between the trials and groups. **(C)** Mean RMI values of AU and AI animals from trial 6 and 10. The data was tested for significant differences using a Welch’s *t*-test (*n* = 8–10/group). Data are shown as mean with SD. AU, aged unimpaired; AI, aged impaired; RMI, reference memory index. A *p*-value of *p* < 0.05 is considered statistically significant, and indicated as * (*p* < 0.05), ** (*p* < 0.01), *** or **** (*p* < 0.001).

The hole-board spatial behavior memory test was performed as previously described (Lubec et al., 2019). In short, a hole-board with the dimension of 1 m × 1 m was constructed of a black plastic board surrounded with translucent Plexiglas walls. Four out of sixteen regularly arranged holes (diameter and depth 7 cm) were baited with food pellets (dustless precision pellets, 45 mg, Bioserv^®^, Flemington, NJ, USA). The pattern of baited holes remained the same during the entire experiment. After handling (15 min per day for four consecutive days) and free exploration time (15 min per day for two consecutive days, access to food pellets), ten training trials within three days were conducted: five trials on day 7, four trials on day 8 and one trial on day 9 (Figure 1A). Each trial lasted 120 s or until all four baited holes were found. The hole visits and removal of the pellets were counted and recorded by video camera. The reference memory index (RMI) was calculated using the formula:


$$R\,M\,I=\frac{\left(f\,i\,r\,s\,t\,v\,i\,s\,i\,t\,o\,f\,b\,a\,i\,t\,e\,d\,h\,o\,l\,e\,s\right)+\left(a\,l\,l\,r\,e\,v\,i\,s\,i\,t\,s\,o\,f\,b\,a\,i\,t\,e\,d\,h\,o\,l\,e\,s\right)}{t\,o\,t\,a\,l\,v\,i\,s\,i\,t\,s\,o\,f\,a\,l\,l\,h\,o\,l\,e\,s}$$

Rats with less than 40 entries in total over the ten trials were excluded from the analysis. Animals with a mean RMI (derived from trial 6 and 10) lower than one standard deviation (SD) compared to the mean RMI of all animals were classified as impaired (AI); rats with a mean RMI higher than one SD compared to the mean RMI of all animals were classified as unimpaired (AU) [see Figure 1B in the original work from Lubec et al. (2019)]. Young male Sprague-Dawley rats (Y, 6 months old) were used as a control.

### 2.3. Animal perfusion and tissue sectioning

Each animal was deeply anesthetized with barbiturate pentobarbital sodium (40–60 mg/kg, intraperitoneal injection) and perfused intracardially with 1.5% paraformaldehyde (PFA) in 0.1 M phosphate buffered saline (PBS, pH = 7.5). The brains were extracted, post-fixed for 1 h in 1.5% PFA solution and cryoprotected in 30% sucrose in PBS at 4°C. When the brain hemispheres were completely soaked with sucrose, they were cut in 40 μm sagittal brain sections using a sliding microtome (Leica, SM2000R). Thereby hemispheres were divided in representative 10th of the brain. Sections were stored at −20°C in 1 ml cryoprotection solution (CPS containing: glycerin, 0.2 M sodium phosphate buffer, ddH_2_O and ethylene glycol at equal proportions).

### 2.4. Fluorescence immunohistochemistry (IHC)

For immunohistochemical analysis of the brain, five randomly chosen brains per group (Y, AU, and AI) were used. Immunohistochemistry was performed free-floating as previously described (Marschallinger et al., 2015; Unger et al., 2020). In short, brain slices were washed 3 × 10 min in 1 × PBS to get rid of excessive cryoprotection solution. Afterward antigen retrieval was performed in pre-cooked 10 mM citrate buffer (pH = 6.0) for 20 min to retrieve the antigen binding sites. This was followed by 3 × 5 min washes with 1 × PBS. To block unspecific binding sites, slices were incubated for 1 h in blocking solution (1% BSA, 0.2% fish skin gelatin and 0.1% Tween in 1 × PBS, all from Sigma-Aldrich, Vienna, Austria). Brain slices were incubated over night at room temperature (RT) with the following primary antibodies: goat anti-FLAP (1:500, Novus Biologicals, Dublin, Ireland, NB300-891), rabbit anti-5-Lox (1:100, Abcam, Amsterdam, Netherlands, ab39347), goat anti-Iba1 (1:500, Abcam, Amsterdam, Netherlands, ab5076), goat anti-Iba1 (1:500, Abcam, Amsterdam, Netherlands, ab107159), rabbit anti-Iba1 (1:500, Abcam, Amsterdam, Netherlands, ab178846), mouse anti-NeuN (1:500, Millipore, Darmstadt, Germany, MAB377).

On the next day, the sections were washed, followed by the incubation with the following secondary antibodies for 4 h: donkey anti-rabbit Alexa Fluor 568 (Invitrogen, Vienna, Austria, A10042), donkey anti-mouse Alexa Fluor 568 (Invitrogen, Vienna, Austria, A10037), donkey anti-goat Alexa Fluor 488 (Invitrogen, Vienna, Austria, A11055), donkey anti-mouse Alexa Fluor 647 (Jackson ImmunoReasearch, Ely, UK, 715605151), donkey anti-rabbit Alexa Fluor 647 (Invitrogen, Vienna, Austria, A31573) (all 1:1000). Additionally, the nucleus dye 4′-6′-Diamidin-2-phenylindol (DAPI 1 mg/ml, 1:2000) was added to stain the cell nuclei. Afterward, the slices were extensively washed (4 × 15 min) in 1 × PBS and auto fluorescence was quenched by incubation with 0.2 % Sudan black (Sigma-Aldrich, Vienna, Austria, in 70% Ethanol, Carl Roth) for 2 min (Schnell et al., 1999). Sections were additionally washed 4 × 5 min in 1 × PBS to remove excessive Sudan black solution. Finally, sections were mounted on microscopy glass slides (Superfrost Plus, Thermo Scientific, Vienna, Austria) and cover-slipped semi-dry with ProLong Gold Antifade Mountant (Life Technologies, Vienna, Austria). To exclude signals from unspecific secondary antibody binding, the primary antibodies were omitted as respective negative controls.

### 2.5. Microscopy and image processing

For the quantitative and qualitative analysis of microglia cells and the immunohistochemical analysis of 5-Lox, confocal microscopy images were taken with the Confocal Laser Scanning Microscope (LSM 700, Zeiss). For the analysis of 5-Lox expression and microglia cell numbers, confocal z-stacks were taken with the ZEN 2011 (black edition) software (Zeiss, version 3.3). Fluorescence images of three different brain slices were taken from five Y, AU, and AI animals per group (*n* = 5/group). Images with 20× and 63× magnification and 0.5 zoom were taken, the obtained z-stack was then combined and merged as maximum intensity projection. For each animal we used four representative areas of the brain that correspond to learning and memory, the hippocampus and the cortex. For all obtained images, the same microscope settings were used, pre-defined on a randomly picked animal of the AI group. The different brain areas were localized by eye.

All confocal images were edited and processed with the ZEN 2012 (blue edition) software (Zeiss, version 3.3), ImageJ (Fiji), Microsoft PowerPoint (2016) and for 3 dimensional (3D) image analysis and co-localization analysis the IMARIS software (version 9.1.2, Bitplane) was used.

### 2.6. Quantification of immunohistochemical data

#### 2.6.1. Quantification of Iba1^+^/5-Lox^+^ and Iba1^+^/5-Lox^–^ cell numbers

Microscopic images from 3 different brain slices per animal with a total of five animals per group (*n* = 5/group) were taken. All images were obtained at the same magnification (20 x, zoom 0.5) and with the same microscopic settings (e.g., laser intensities). To assess neuroinflammation the number of DAPI / Iba1 positive stained microglia cells (Iba1^+^) was manually counted using the Fiji software with the help of the cell counter plug in. Additionally, the number of microglia positive for 5-Lox (Iba1^+^/5-Lox^+^) was assessed. All microglia that did not show 5-Lox staining were classified as 5-Lox negative microglia cells (Iba1^+^/5-Lox^–^). Percentages of Iba1^+^/5-Lox^+^ and Iba1^+^/5-Lox^–^ cell numbers of the total Iba1^+^ cell counts were calculated.

#### 2.6.2. Quantification of percentage (%) 5-Lox and FLAP staining

The overall percentages of the different stainings (5-Lox, FLAP) for each image was calculated for three different brain slices per animal with a total of five animals per group (*n* = 5/group). All images were taken with the same microscope settings and magnification (20 x, zoom 0.5). The confocal z-stacks were analyzed with IMARIS (Imaris x64 9.1.2., Bitplane), the volume (μm^3^) of the respective staining was displayed as 3D surface and the threshold was set manually for each image. The 3D surface tool of IMARIS (Imaris x64 9.1.2., Bitplane) analyzes the number and volume (μm^3^) of each antibody stained positive particles. To calculate the percentage (%) of staining in respect to the total volume of the image (μm^3^) the sum of all positive particle volumes (μm^3^) per image was used, respectively for each antibody staining. The mean of all three slices per animal (*n* = 5 animals/group) for each brain region was calculated.

#### 2.6.3. Co-localization of 5-Lox and FLAP staining with NeuN or Iba1

The co-localization of 5-Lox and FLAP with either NeuN or Iba1 was analyzed in three different slices per animal (*n* = 5/group). All images were taken with the same setting and magnification (20×, zoom 0.5). For analysis of the co-localization the “Coloc” tool of IMARIS (Imaris x64 9.1.2., Bitplane) was used. Either NeuN or Iba1 was set as channel A, and 5-Lox or FLAP was set to be channel B. For each image, the threshold for both channels was set manually and the value for “% of dataset co-localized” was used for quantification. The percent of dataset co-localized shows the total amount of voxel in the images that are co-localized with both channels (overlap) in respect to the overall image. The mean of all three slices per animal (*n* = 5/group) for each brain region was calculated and results show again data for the hippocampus or cortex.

### 2.7. Statistical analysis

All statistical analysis was done with Graph Pad Prism software (Version 9, Graph Pad Software Inc., San Diego, California, USA). We refrained from testing for normality due to the small sample size of *n* = 5 (Norman, 2010). For group comparisons between AU and AI, an unpaired *t*-test or Welch’s *t*-test was performed depending on equality of variance or as otherwise indicated in the figure legends. For comparison of more than two groups (Y, AU, AI), an ordinary one-way analysis of variance (ANOVA) was used with Tukey’s *post-hoc* correction for multiple comparisons or as otherwise indicated in the figure legends. To compare the RMI in the different trials of AU and AI animals a two-way ANOVA with repeated measures (RM) was used with Sidak’s correction for multiple comparisons. Data is shown as median and 25th to 75th percentiles, and whiskers indicate the minimum and maximum values (as indicated in respective figure legends). A *p*-value of *p* < 0.05 is considered statistically significant, and indicated as * (*p* < 0.05), ** (*p* < 0.01), *** or **** (*p* < 0.001). To identify outliers a Grubb’s test was performed. In the histological analysis, where sample size was low, significance testing is less important or not even required. Therefore, we used effect sizes, focusing on absolute differences in measured variables, expressed as η^2^, which is more suited for interpretation. Here, 0.01 < η^2^ < 0.06 are considered small effects, 0.06 < η^2^< 0.14 are considered medium effects, and η^2^> 0.14 are considered as large effects.

### 2.8. Ethical approval

The study was carried out in accordance with the recommendations of the European Communities Council Directive of 24 November 1986 (86/609/EEC) evaluated by the ethics committee of the Medical University of Vienna, Austria (Lubec et al., 2019).

## 3. Results

### 3.1. Aging rats develop into cognitively impaired and cognitively unimpaired traits

Hole-board, a food-motivated spatial learning and memory task, was used to evaluate the effect of aging on spatial learning and memory in a big cohort of 22–24-months-old male Sprague–Dawley rats. The experimental protocol is presented in Figure 1A. Rats characterized as either superior (AU) or inferior (AI) based on their mean reference memory indices (RMI) derived from trial 6 and 10 of retrieval phases were randomly selected from AU and AI from a larger cohort of 160 rats (Lubec et al., 2019). Rats characterized as AU performed significantly better compared to AI (Two-way RM-ANOVA, *p* < 0.001; Figures 1B, C). Trial-by-trial analysis shows that RMIs of AU animals increased steadily over the course of 10 trials, indicating learning, whereas AI animals did not learn the task (Figure 1B).

### 3.2. Increased 5-Lox expression in brains of aged and cognitively impaired rats

**FIGURE 2:**
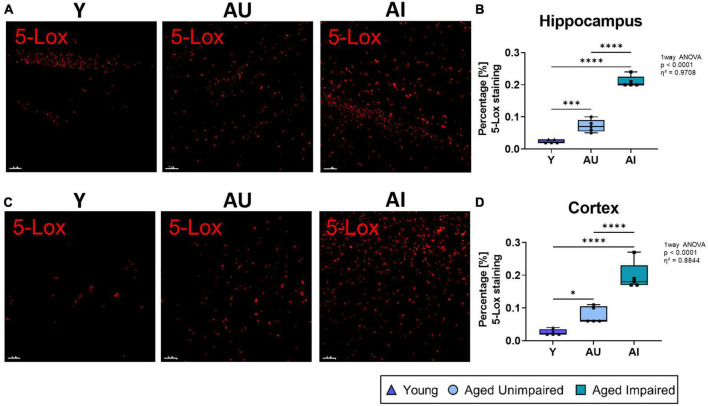
Immunohistochemistry analysis and 3D structures 5-Lox staining in the hippocampus and cortex of Y, AU, AI rats. **(A)** Representative images of 5-Lox 3D structure in the hippocampal DG region of all three groups. **(B)** Percentage of 5-Lox staining was significantly increased in the hippocampus of old rats and AI animals had the highest amount of 5-Lox staining compared to AU. **(C)** Representative images of 5-Lox 3D structure in the prefrontal cortex of Y, AU, and AI animals. **(D)** Percentage of 5-Lox staining was significantly increased in the cortex of old animals with the highest amount of 5-Lox staining in AI rats compared to AU. All images were processed with the surface tool of the IMARIS (Bitplane) software, and all data were analyzed using an ordinary One-way ANOVA with Tukey’s multiple comparisons test. Box plots show median and 25th to 75th percentiles and whiskers indicate the minimum and maximum values. Y = young, AU = aged unimpaired, AI = aged impaired. Scale bar: 50 μm. η^2^ indicates large effects. A *p*-value of *p* < 0.05 is considered statistically significant, and indicated as * (*p* < 0.05), ** (*p* < 0.01), *** or **** (*p* < 0.001).

To address the question whether an elevated LT system might relate to the age-related cognitive decline and more importantly, to cognitive heterogeneity in aging, we investigated the brains of Y, AU and AI rats for expression of 5-Lox and FLAP using immunohistochemistry. The overall percentage of 5-Lox staining was quantified in the hippocampus (Figures 2A, B). As expected in old rats (AU and AI), the percentage of 5-Lox staining was significantly higher compared to young animals, which showed very little 5-Lox signals (overall effect size η^2^ = 0.9708) (Figure 2B). Most surprisingly, in the brains of AI rats the percentage of 5-Lox staining was significantly higher compared to AU animals and was the highest of all groups (Figure 2B). Similar results were obtained in the cortex (effect size η^2^ = 0.8844) (Figures 2C, D).

### 3.3. Increased number of 5-Lox^+^ microglia in AI compared to AU rats

**FIGURE 3:**
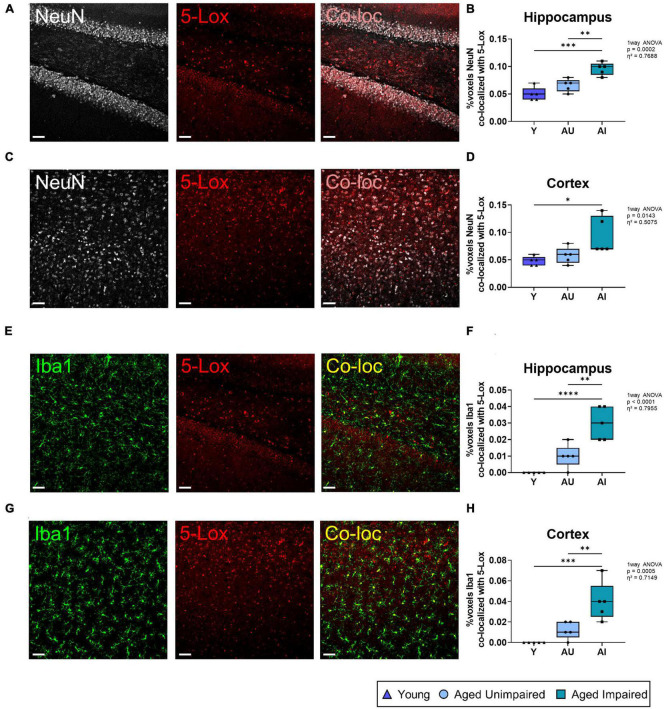
Co-localization analysis of 5-Lox staining with neurons (NeuN) and microglia (Iba1). **(A)** Representative images of NeuN and 5-Lox and the resulting co-localization signal in the hippocampus. **(B)** Co-localization of NeuN with 5-Lox was increased in old (AU and AI) rat brains compared to Y. **(C)** Representative images of NeuN and 5-Lox and the resulting co-localization signal in the cortex. **(D)** Percentage of co-localization between NeuN and 5-Lox was increased in the cortex of AU and AI animals compared to young. **(E)** Representative images of an AI animal with 3D structure for the Iba1 and 5-Lox staining and the resulting co-localization signal in the hippocampus. **(F)** In the hippocampus of AI rats there was significantly more co-localization of Iba1 with 5-Lox compared to Y and AU animals. **(G)** Representative images of an AI rat with 3D structure for Iba1 and 5-Lox staining and the resulting co-localization signal in the cortex. **(H)** Co-localization of Iba1 with 5-Lox was significantly increased with age (Y vs. AU and AI) and cognitive decline (AU vs. AI) in the cortex. All images were processed with the Co-loc tool of the IMARIS (Bitplane) software and all data were analyzed using an ordinary One-way ANOVA with Tukey’s multiple comparisons test. Box plots show median and 25th to 75th percentiles and whiskers indicate the minimum and maximum values. Y = young, AU = aged unimpaired, AI = aged impaired. Scale bar: 50 μm (all images). η^2^ indicates large effects. A *p*-value of *p* < 0.05 is considered statistically significant, and indicated as * (*p* < 0.05), ** (*p* < 0.01), *** or **** (*p* < 0.001).

5-Lox is known to be expressed in the brain predominantly in microglia, although neurons show also some 5-Lox immunoreactivity (Zhang et al., 2006; Michael et al., 2020; and reviewed in Michael et al., 2018). To evaluate if the herein observed increase in 5-Lox expression in AI rat brains derives from neurons or microglia, we performed co-localization analysis of 5-Lox staining with the mature neuron marker NeuN and the microglial marker Iba1 (Figure 3). First, the percentage of 5-Lox overlapping with neurons was quantified by co-localization analysis of 5-Lox with NeuN staining in the hippocampus (Figure 3A). In AI animals the percentage of co-localization between 5-Lox and NeuN staining was significantly higher compared to AU as well as to young rats (effect size η^2^ = 0.7688) (Figure 3B). Similar results were obtained from the cortex (effect size η^2^ = 0.5075) (Figures 3C, D). Next, the percentage of 5-Lox staining that overlapped with microglia (Figures 3E, G) was quantified in the hippocampus (Figure 3F) and the cortex (Figure 3H) by analyzing the percentage of dataset of 5-Lox that co-localized with the Iba1 staining. Remarkably, in the hippocampus of AI brains the percentage of 5-Lox staining co-localizing with Iba1 was significantly higher compared to AU and young animals (effect size η^2^ = 0.7955). In young animals there was barely any co-localization of 5-Lox with Iba1 detected (Figure 3F). Similar data were obtained from the cortex (effect size η^2^ = 0.7149) (Figure 3H). This let us conclude that the observed difference in 5-Lox in AI compared to AU animals derives mainly from microglia cells and less from neurons.

**FIGURE 4:**
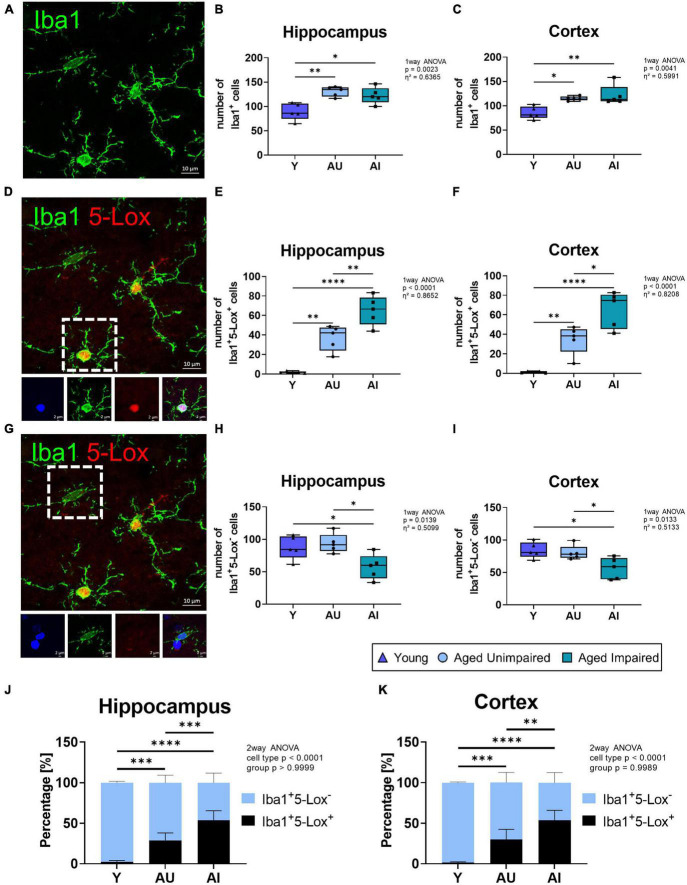
Analysis of microglia (Iba1^+^), 5-Lox positive (Iba1^+^5-Lox^+^) and 5-Lox negative (Iba1^+^5-Lox^–^) microglia cell numbers in the brain of Y, AU, and AI animals. **(A)** Representative image of microglia cells at 63 x magnification. **(B)** Quantification of microglia cell numbers in the hippocampus and cortex **(C)** of Y, AU, and AI animals (*n* = 5/group). Microglia numbers significantly increase with age. **(D)** Representative image of Iba1^+^5-Lox^+^ microglia (insert) at 63× magnification. Inserts represent Iba1^+^5-Lox^+^ microglia. **(E)** Quantification of Iba1^+^5-Lox^+^ microglia in the hippocampus and cortex **(F)** (*n* = 5/group). Double positive cells increase significantly with age, and rise even further in the AI group compared to the AU animals in both brain regions. **(G)** Representative image showing an Iba1^+^5-Lox^–^ microglia cell (insert). Inserts represent Iba1^+^5-Lox^–^ microglia. **(H)** Quantification of Iba1^+^5-Lox^–^ microglia in the hippocampus and cortex **(I)** of Y, AU, and AI animals (*n* = 5/group). The amount of Iba-1^+^5-Lox^–^ microglia is significantly decreased in the AI group compared to the young as well as to the AU group. **(J)** Percentage of 5-Lox^+^ and 5-Lox^–^ microglia in the overall Iba1^+^ microglia cell counts in the hippocampus and cortex **(K)**. Data was tested using an ordinary One-way ANOVA with Tukey’s multiple comparison test **(B,C,E,F,H,I)** and Two-way ANOVA with Sidak’s multiple comparison test **(J,K)**. Box plots show median and 25th to 75th percentiles and whiskers indicate the minimum and maximum values, for panels **(J,K)** data are as mean with SD. Scale bar: 10 μm (all images) and 2 μm (inserts). Y = young, AU = aged unimpaired, AI = aged impaired. η^2^ indicates large effects. A *p*-value of *p* < 0.05 is considered statistically significant, and indicated as * (*p* < 0.05), ** (*p* < 0.01), *** or **** (*p* < 0.001).

To further support the hypothesis of enhanced 5-Lox expression in microglia of AI brains, a more detailed analysis was performed. Therefore, the number of overall Iba1^+^ cells (Figure 4A), Iba1^+^ cells positive for 5-Lox (Iba1^+^5-Lox^+^ cells, Figure 4D) or Iba1^+^ cells negative for 5-Lox (Iba1^+^5-Lox^–^ cells, Figure 4G) was quantified in hippocampal and cortical brain regions. In the hippocampus and the cortex of old rat brains (AU and AI) the number of Iba1^+^ cells were significantly increased compared to young animals (effect size hippocampus η^2^ = 0.6365, effect size cortex η^2^ = 0.5991) (Figures 4B, C). However, the number of Iba1^+^ cells was similar in AI and AU animals (Figures 4B, C). In aged animals (AU and AI) the number of Iba1^+^5-Lox^+^ cells was significantly higher compared to young rats, which barely showed any Iba1^+^5-Lox^+^ cells. Most surprisingly, in the brains of AI animals the number of Iba1^+^5-Lox^+^ cells was significantly higher compared to AU rats (effect size hippocampus η^2^ = 0.8652, effect size cortex η^2^ = 0.8208) (Figures 4E, F). Analysis of the number of Iba1^+^5-Lox^–^ cells (Figure 4G) revealed no difference in the number of cells between young rats and AU animals (Figures 4H, I). Remarkably, the number of Iba1^+^5-Lox^–^ cells was significantly lower in AI animals compared to AU and to young rats in both brain regions (effect size hippocampus η^2^ = 0.5099, effect size cortex η^2^ = 0.5133) (Figures 4H, I). This indicates an overall shift in the percentage of microglia that express 5-Lox, which we confirmed by calculating the percentages of Iba1^+^5-Lox^+^ and Iba1^+^5-Lox^–^ cells in the total Iba1^+^ cell fraction in the hippocampus and cortex of the animals (Figures 4J, K). In summary, there is a shift in the 5-Lox expression in microglia cells with age and even more pronounced an upregulation of 5-Lox expression in microglia cells in cognitively impaired animals.

### 3.4. Increased FLAP expression co-localizing with microglia in AI rats

**FIGURE 5:**
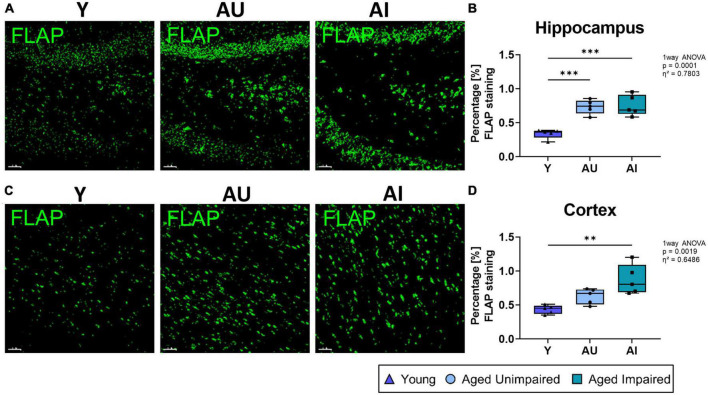
Quantification of overall FLAP staining in the brain. **(A)** Representative images of 3D structure for the FLAP staining in the hippocampus of Y, AU, and AI animals. **(B)** Quantification of percentage FLAP staining in the hippocampus shows a significant increase in the AU and AI group compared to the young animals. **(C)** Representative images of 3D structure for the FLAP staining in the cortex of Y, AU, and AI animals. **(D)** Quantification of percentage FLAP staining in the cortex. Here we also see an increase in FLAP staining with age, which is significant in the AI group. All images were analyzed with the IMARIS (Bitplane) software and all data were analyzed using an ordinary One-way ANOVA with Tukey’s multiple comparisons test. Box plots show median and 25th to 75th percentiles and whiskers indicate the minimum and maximum values. Y = young, AU = aged unimpaired, AI = aged impaired. Scale bar: 50 μm **(A,C)**. η^2^ indicates large effects. A *p*-value of *p* < 0.05 is considered statistically significant, and indicated as * (*p* < 0.05), ** (*p* < 0.01), *** or **** (*p* < 0.001).

Next, we investigated the expression of the 5-Lox activating protein, FLAP, in the hippocampus and cortex (Figures 5A, C). First we quantified the percentage staining of overall FLAP and observed increased expression of FLAP in the hippocampus of old (AU and AI) compared to young (Y) animals (effect size η^2^ = 0.7803) (Figure 5B), whereas in the cortex, although the effect size was η^2^ = 0.6486, only a significant increase was observed between the young and the AI animals (Figure 5D). However, there was no significant difference in FLAP staining between AI rats compared to AU animals in either region.

**FIGURE 6:**
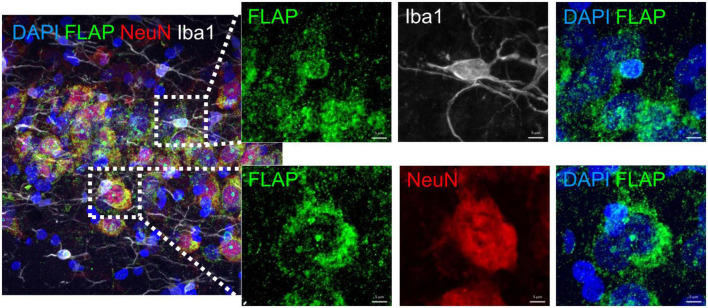
Immunohistochemistry analysis of FLAP staining with microglia (Iba1) and neurons (NeuN). Representative images revealing FLAP staining at sites of the nuclear membrane in microglia (Iba1) and in- and outside the nucleus of neurons (NeuN). Dapi was used as nucleus stain. Scale bar: 20 μm, 5 μm.

**FIGURE 7:**
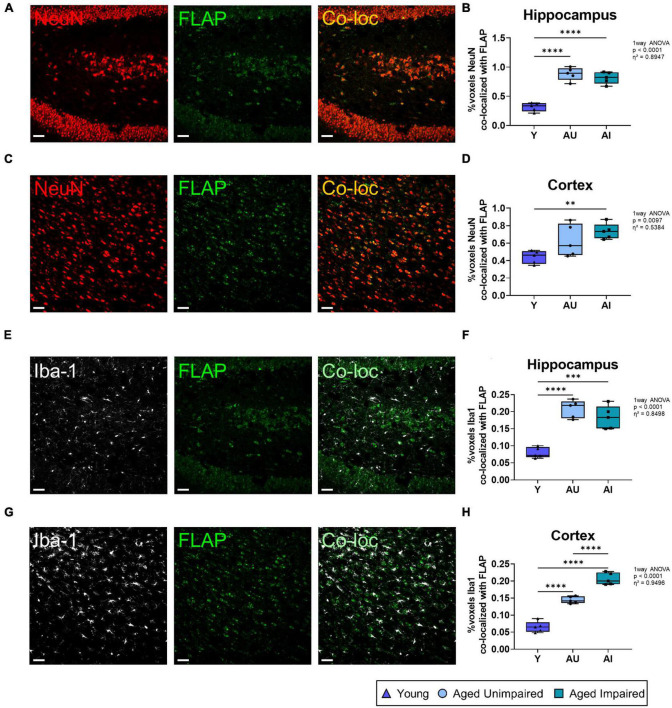
Quantification of co-localization between FLAP staining and neurons (NeuN) or microglia (Iba1). **(A)** Representative images of co-loc of FLAP with NeuN in the hippocampus. **(B)** Quantification of FLAP and NeuN co-loc signals in the hippocampus. The co-localization increases with age in both aged groups compared to the young group. **(C)** Representative images of co-loc of FLAP with NeuN in the cortex. **(D)** Quantification of FLAP and NeuN co-loc signals in the cortex. The co-localization is significantly increased in the AI group. **(E)** Representative images of the co-localization signal of FLAP with Iba1 in the hippocampus of Y, AU, and AI animals. **(F)** Quantification of FLAP and Iba1 co-loc signals in the hippocampus. The co-localization signal is highly increased in both aged groups compared to the young group. **(G)** Representative images of the co-loc of FLAP with Iba1 in the cortex. **(H)** Quantification of FLAP and Iba1 co-loc signals in the cortex. Significant increases in the co-localization signal is seen in both aged groups compared to the young animals. There is also a significant increase in the AI group compared to the AU group. All images were processed with the Co-loc tool of the IMARIS (Bitplane) software and all data were analyzed using an ordinary One-way ANOVA with Tukey’s multiple comparisons test. Box plots show median and 25th to 75th percentiles and whiskers indicate the minimum and maximum values. Y = young, AU = aged unimpaired, AI = aged impaired. Scale bar: 50 μm **(A,C,E,G)**. η^2^ indicates large effects. A *p*-value of *p* < 0.05 is considered statistically significant, and indicated as * (*p* < 0.05), ** (*p* < 0.01), *** or **** (*p* < 0.001).

In human and in mouse brains we recently demonstrated that FLAP was exclusively detected in microglia cells but not in neurons (Michael et al., 2020). However, in rat brains we detected FLAP not only in microglia cells but also in neurons, mostly localized in and around the cell nucleus (Figure 6). This is in line with one other study showing neuronal FLAP expression on rat brain tissue (Lammers et al., 1996). We therefore further analyzed the co-localization of FLAP staining with the mature neuron marker NeuN and the microglia marker Iba1, to determine which cell type contributes to the increased FLAP expression (Figure 7). First, we quantified the percentage of FLAP staining co-localizing with NeuN in the hippocampus (Figures 7A, B). In aged animals (AU and AI) the percentage of co-localization between FLAP and NeuN was increased compared to young animals but there was no difference between AU and AI rats (effect size η^2^ = 0.8947) (Figure 7B). Similar data were obtained from the cortex (effect size η^2^ = 0.5384) (Figures 7C, D). Next, we quantified the percentage of FLAP staining co-localizing with Iba1 in the hippocampus (Figures 7E, F). Aged animals (AU and AI) showed increased percentages of co-localization of FLAP with Iba1 but no differences between AI and AU animals in the hippocampus (effect size η^2^ = 0.8498) (Figure 7F). However, in the cortex not only the increase between the age groups was observed, but also significantly higher percentages of co-localization between FLAP and Iba1 were detected in AI rats compared to AU animals (effect size η^2^ = 0.9496) (Figures 7G, H).

Taken together, FLAP expression was increased in the brain of aged compared to young animals. In microglia cells the FLAP expression in the cortex was additionally increased in AI compared to AU animals, which suggests that FLAP expressing microglia contribute to a cognitive decline.

## 4. Discussion

### 4.1. 5-Lox^+^ microglia: a subpopulation determining cognitive impairment in aging?

One hallmark of brain aging is neuroinflammation that is mainly driven by glial cells in particular microglia, the brain resident immune cells. Those are primed to an altered inflammatory profile releasing pro-inflammatory molecules in the old brain (Sierra et al., 2007 and reviewed in Dilger and Johnson, 2008; Mosher and Wyss-Coray, 2014), and are thereby mainly responsible for neuroinflammation (reviewed in Kettenmann et al., 2011). The contribution of neuroinflammation but also of microglia dysfunction to disease progression is already a well-established hallmark for various neurodegenerative diseases, such as AD (reviewed in Mosher and Wyss-Coray, 2014; Angelova and Brown, 2019; Hemonnot et al., 2019). Alterations in microglia in the context of physiological aging are less evident, however, recent studies demonstrate, that microglia function and microglia phenotypes are heavily altered in the aged brain (Sierra et al., 2007; Marschallinger et al., 2020b), which leads to a heterogeneous population of microglia cells during aging (reviewed in Mosher and Wyss-Coray, 2014; Delage et al., 2021). For example, we previously identified lipid-droplet accumulating microglia (LDAM) as a subpopulation of microglia cells that are increasingly present in the aged human and mouse brain. Those microglia cells are associated with a dysfunctional and pro-inflammatory state in the aged brain (Marschallinger et al., 2020b). Besides these LDAMs, microglia subpopulations such as disease-associated microglia (DAM) (Keren-Shaul et al., 2017) and AD-associated microglia cells (Wang et al., 2015) have been identified in the neurodegenerated brain. Our herein described data on 5-Lox^+^ microglia cells in aged cognitively impaired rats is therefore additional evidence for microglia heterogeneity and for microglia subpopulations, which contribute to the age-related cognitive decline. Microglia, which express 5-Lox and FLAP can be considered as the main producers of LTs within the brain, thus are potentially highly involved in LT biosynthesis (Michael et al., 2020). Previously, we demonstrated that microglia depletion in adult WT and transgenic AD mice resulted in massive downregulation of various parts of the LT signaling pathway. Microglia depleted brains showed a massive reduction in 5-Lox, FLAP, and CYSLTR1 on mRNA and/or protein level (Michael et al., 2020). We concluded from this work that microglia cells harbor key elements of the LT biosynthesis pathway and are therefore a potential major source of LTs in the brain. This is also seen in an open database of the cell-specific transcriptome of a broad spectrum of cells of the human aged healthy and Alzheimer’s disease brains (Yang et al., 2022). Here, *Alox5* (the gene encoding 5-Lox) and *Alox5AP* (the gene encoding FLAP) expression is predominantly expressed in microglia, in particular in Alzheimer’s disease brains. It is very likely that 5-Lox^+^ microglia thereby represent a specific subpopulation of microglia in the aged brain, however, the exact function and phenotype of these cells in the aged brain would need further in depth investigations (reviewed in Delage et al., 2021).

### 4.2. Leukotriene signaling: a therapeutic target to restore brain structure and function in aging and in neurodegenerative diseases

Leukotriene signaling has been identified as a promising target to restore structure and function in neurodegenerative diseases. For example, 5-Lox and FLAP expression is increased in AD, in particular in microglia cells (Michael et al., 2020; Michael et al., 2021; Yang et al., 2022). Furthermore, experimental overexpression of 5-Lox leads to a decline in cognition, decreased memory and to an increased activation of microglia (Vagnozzi et al., 2018). Previous studies have shown, that treatment with the leukotriene inhibitor MTK significantly reduced the percentages of 5-Lox^+^ microglia cells in the brain of 5xFAD transgenic mice (Michael et al., 2021). Similarly, Montelukast treatment improved cognition in animal models of Lewy body dementia (Marschallinger et al., 2020a) and stroke (Gelosa et al., 2019). Besides in neurodegenerative diseases, leukotriene signaling has been identified as a target in brain aging. For example, treatment with the LT receptor antagonist Montelukast reduced neuroinflammation and restored learning and memory in aged rats (Marschallinger et al., 2015). In the present study, we could show that leukotriene signaling is not only changed in neurodegenerative diseases, but is also altered during normal healthy aging, possibly contributing to cognitive heterogeneity. We could confirm that the overall 5-Lox and FLAP expression in microglia cells and neurons was significantly increased in aged animals. This is in line with previous studies showing upregulated 5-Lox mRNA and protein levels in the hippocampus of old mice (Chinnici et al., 2007) and increased 5-Lox protein expression in the brain of old rats (Uz et al., 1998). However, there is inconsistency on the cell type responsible for the age-related increase in 5-Lox immunoreactivity. In the study by Uz et al. (1998) the age-related increase in 5-Lox expression derives from pyramidal neurons or GFAP positive cells. In our study, we confirmed the age-related increase in neuronal 5-Lox expression, however, we do not see differences in neuronal 5-Lox expression in AI compared to AU animals. We clearly demonstrate increased 5-Lox immunoreactivity in microglia and increased numbers of 5-Lox^+^ microglia in the brain of aged animals. Remarkably, more detailed analysis of AU and AI rat brains revealed that this increase was even exceeded in microglia of cognitively declined animals. To our knowledge this has so far not been demonstrated in any rodent model for aging, in which animals were classified according to their cognitive performance.

This underlines the involvement of LTs in cognitive decline, and supports the hypothesis that leukotriene inhibition could be used as a therapeutic target during age related cognitive decline and in neurodegenerative diseases. However, the possible beneficial effects of LT signaling blockage on the brain and the direct effects of MTK on the LT signaling pathway involved cell types should be assessed in more detail in future experiments.

### 4.3. Leukotrienes to distinguish cognitive heterogeneity

During natural, healthy aging loss of cognition is often part of the aging process, however, the decline in cognition is very heterogenic (Gallagher and Rapp, 1997; Gallagher et al., 2006; Lubec et al., 2019). Cognitive heterogeneity is frequently studied and well described in the field of psychology (Ylikoski et al., 1999; Mungas et al., 2010; Hayden et al., 2011), however, the molecular mechanisms are mostly unclear. Several pathways have been shown to be differentially regulated in cognitive impairment, such as pathways involving oxidative stress (Lubec et al., 2019) or neuroinflammation (Farso et al., 2013). Previous studies have shown that aged cognitive impaired and aged cognitive unimpaired rats have a different lipid content in the brain, i.e., impaired animal have a reduced phosphatidylethanolamine (LPE) content (Wackerlig et al., 2020). Interestingly, restoration of the LPE content reduces arachidonic acid production and improves cognition (Villamil-Ortiz et al., 2016), by possibly reducing the LT load in the brain. This already suggests the involvement of leukotrienes in the cognitive heterogeneity in aging. In the present study we focused on the cognitive heterogeneity in aging in the context of leukotriene signaling. We could demonstrate that an increased 5-Lox expression, especially in microglia cells, is associated with a decreased memory function in aged rats and vice versa that low expression of 5-Lox in microglia associates with better memory scores. These findings suggest, that LTs could possibly be used to distinguish between different cognitive states in aging, even before the onset of neurodegenerative diseases.

### 4.4. 5-Lox: a potential biomarker for cognitive decline?

The present work suggests that LTs and the leukotriene signaling pathway might be possible candidates for biomarkers to predict cognitive decline in aging. Overexpression of 5-Lox has been shown to worsen memory, increase microglia and astrocyte activation and significantly impact synaptic pathology (Vagnozzi et al., 2018), making 5-Lox a potential indicator for cognitive decline. Vice versa, blocking the LT signaling pathway has beneficial effects leading to restoration of memory and cognitive functions (Marschallinger et al., 2015). Additionally, it was shown that unresolved neuroinflammation after surgery, linked to high levels of leukotrienes, is connected to cognitive decline (Su et al., 2013), underlining the possibility to use LTs as a biomarker for cognitive decline. Our data also associates 5-Lox expression in microglia with cognitive decline, supporting the idea to use the LT signaling pathway not only as a therapeutic target for AD but also to use it already for the prediction of cognitive decline in early stages. In summary, 5-Lox might be used as a potential biomarker for cognitive decline, which will be highly beneficial for diagnostic and therapeutic purposes as well as for prediction of cognitive decline. In the field of asthma, urinary LTs predict the severity and outcome of asthma (Szefler et al., 2012; Hoffman and Rabinovitch, 2018) as well as the responsiveness to leukotriene receptor inhibitors such as MTK (Hoffman and Rabinovitch, 2018). Whether this can be used to translate into a biomarker for cognitive decline in aging needs to be shown (Su et al., 2013; Furuyashiki et al., 2019). An interesting approach might be 5-Lox expression in peripheral blood mononuclear cells (PBMCs), which are easily accessible and known to share some of the biochemical environment of neurons (Dangond and Gullans, 1998; de Ruijter et al., 2003). Indeed, it has been demonstrated that AD patients have increased levels of 5-Lox expression in PBMCs (Di Francesco et al., 2013).

### 4.5. Limitations

We are aware, that our study has limitations, such as the low number of animals analyzed, giving less importance to the significance values. Considering the work as an exploratory study allows the focus on the effect sizes showing that despite the low number of animals, we do observe large effects. Nevertheless, confirmatory studies using a higher n are needed to validate our results. The study would also benefit from the use of other methods, such as protein or RNA analysis.

In the present study, we examined the two extremes in terms of cognitive performance. Of course, it would be interesting to investigate the 5-Lox and FLAP expression in the brains of aged animals with an average cognitive ability. Unfortunately, we did not have access to the brains of such “average performers.” The aim of the study was to focus on the underlying molecular cues discriminating extreme good and bad cognitive performers. Thus, we consider our study as a first exploratory investigation in the field, of course creating new questions, which can be hopefully addressed in future studies.

## 5. Conclusion

We graphically summarized our immunohistochemical findings on altered LT signaling components in the aged and cognitively impaired rat brain (Supplementary Figure 1). In the aged brain increased numbers of 5-Lox positive microglia are present with the highest number in cognitively impaired animals. FLAP protein expression is increased in microglia cells of aged and even more pronounced of cognitively impaired animals. We conclude from our results that major compounds of the LT signaling pathway are elevated in aged cognitively impaired brains.

## Data availability statement

The raw data supporting the conclusions of this article will be made available by the authors, without undue reservation.

## Ethics statement

The animal study was reviewed and approved by the Medical University of Vienna.

## Author contributions

HM, JF, MU, and LA drafted the manuscript. JF and HM performed histology, RNA isolation, and gene expression analysis. IA performed histology and helped with sample preparation. MK, AH, JL, JM, and DF performed experiments with animals and/or data analysis. GL, VK, and HH designed and guided behavioral experiments. LA was the principle investigator of the study and was involved in the experimental design and critical revision of the manuscript. All authors contributed to the article and approved the submitted version.

## Supplementary material

Supplementary Figure 1 Alterations of the LT signaling pathway in brains of Y, AU, and AI animals. Brains were immunohistochemically analyzed and revealed alterations in 5-Lox and FLAP protein expression in aged and cognitively impaired animals. Image was created with Biorender.com.
