# Supplementary figures and images for: Leukotriene signaling as molecular correlate for cognitive heterogeneity in aging: an exploratory study
Source: Front Aging Neurosci. 2023 Aug 2;15:1140708. doi: 10.3389/fnagi.2023.1140708 (PMC10433382; doi:10.3389/fnagi.2023.1140708)

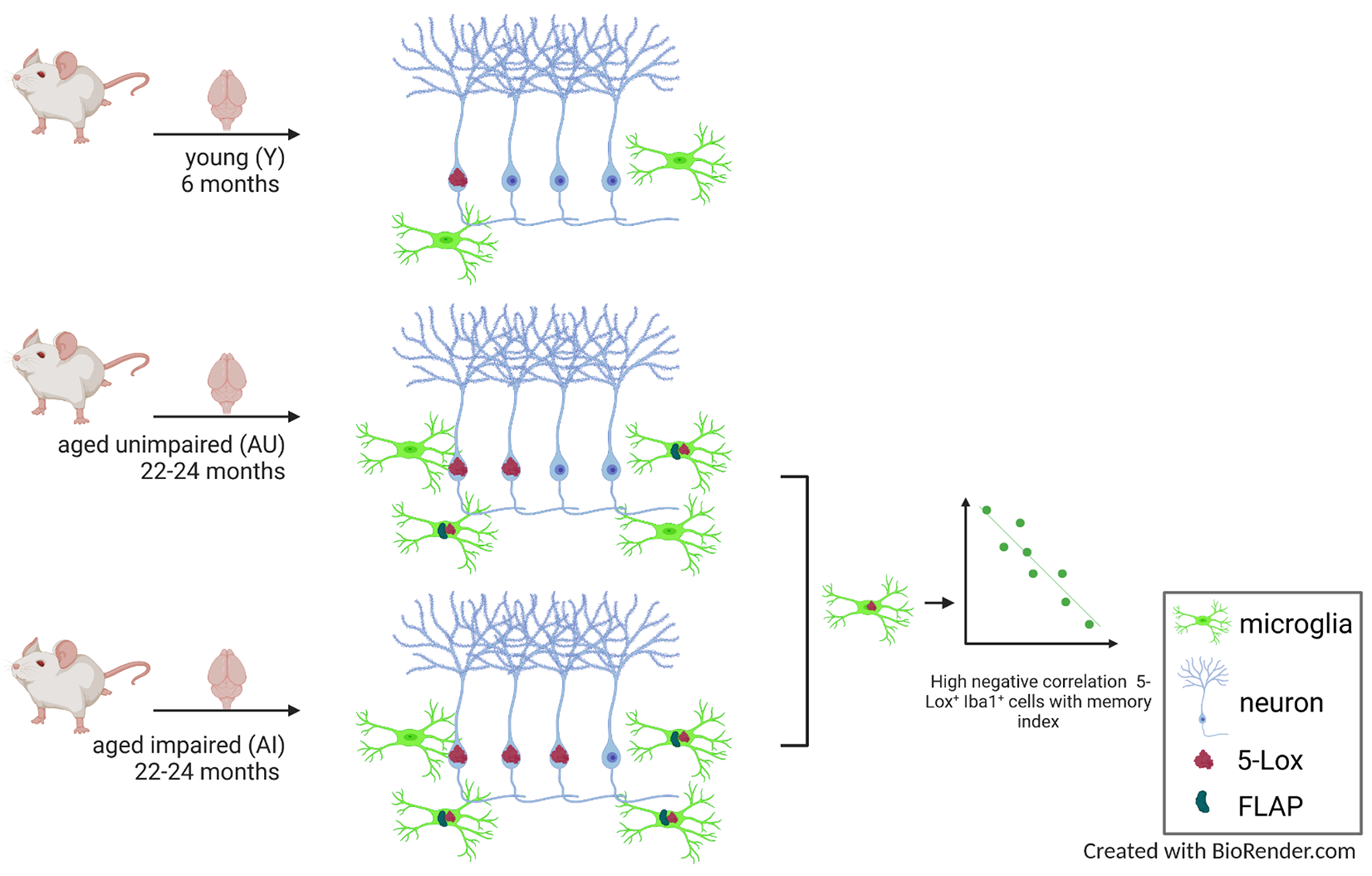

Supplement: Supplementary Figure 1 — Alterations of the LT signaling pathway in brains of Y, AU, and AI animals. Brains were immunohistochemically analyzed and revealed alterations in 5-Lox and FLAP protein expression in aged and cognitively impaired animals. Image was created with Biorender.com. [file Image_1.TIF]
